# Rethinking Aid Allocation: Analysis of Official Development Spending on Modern Pollution Reduction

**DOI:** 10.5334/aogh.2633

**Published:** 2019-11-07

**Authors:** Stephanie Swinehart, Richard Fuller, Rachael Kupka, Marc N. Conte

**Affiliations:** 1 Fordham University, US; 2 Pure Earth, New York, NY, US; 3 Global Alliance on Health and Pollution, New York, NY, US; 4 Department of Economics, Fordham University, US; 5 New York University, New York, NY, US

## Abstract

**Background:**

Modern pollution – pollution attributable to industrialization and urbanization – is responsible for nearly 6 million deaths per year, more than all the deaths from HIV, malaria, and tuberculosis combined; yet it receives comparatively little attention in the international development agenda [1].

**Objective/Methods:**

This study attempts to highlight the funding disparity between select key threats to global health by quantifying the levels of international official development aid (ODA) allocated to reducing pollution’s negative impact on human health using a new metric – dollars spent per death caused by health threat.

**Findings:**

Using only reported ODA spending for 2016, we calculate an average investment of $14/death for modern pollution, compared with $1,250/death for malaria, $190/death for tuberculosis, and $165/death for HIV/AIDS.

**Conclusions:**

Although there are substantive limitations to this analysis, results are sufficient to galvanize action to better monitor and track investments in modern pollution reduction. Donor countries have failed to respond to this urgent public health crisis. Given the severity of its public health burden, there is a critical need for funding to be allocated *specifically* to pollution reduction.

## I. Introduction

Recognition of the impacts of exposure to pollution is growing. In 2017, the *Lancet* Commission on Pollution and Health called pollution “the largest environmental cause of disease and death in the world,” killing 9 million prematurely in 2015 [1]. Ambient air and plastics pollution are frequently featured in the media. In May 2019, pollution was listed as one of the top five direct drivers of change today, with catastrophic impacts for people and the planet [2].

Despite this, quantifying the depth of pollution’s impact on human health and productivity and providing justification for allocation of resources is challenging. Difficulties evaluating the impacts of pollution stem from its multiple sources, its difficulty to monitor, and from isolating the multiple comorbidities that exist from exposure. Industries and governments operate on the incorrect assumption that income today is more valuable than environmental quality tomorrow – discounting the impacts of poor environmental quality on worker productivity, healthcare costs, and economic performance. The *Lancet* Commission estimates that GDP losses from pollution in low- to middle- income countries could be up to 2% per year and account for up to 7% of annual health spending [1]. Additional losses associated with toxic exposure include up to 1.2% of global GDP for childhood lead exposure and occupational productivity losses associated with polluted air [1,3]. The *Lancet* Commission also notes that while deaths associated with traditional pollution (indoor air pollution caused by cooking using wood or other sources, and polluted water) are falling, deaths associated with modern pollution (air, water and soil pollution associated with industrialization and urbanization) are rising at unprecedented levels [1].

Measuring the welfare impacts of pollution is also difficult. Industries of all sizes and types and cities generate excessive levels of pollution, yet the precise impacts are difficult to quantify, and markets do not exist to value these impacts once identified. Environmental accounting can take several forms, ranging from national accounting to measurement through marginal cost and damage functions, but all approaches require substantial effort and expertise not always available to policy makers [4]. Without information about the costs of abatement and the benefits of reducing emissions, it is not possible to identify the optimal areas for investments with the most benefit on human health. Further, in some industries, especially the informal sector, a lack of incentive to improve practices may be present due to risk or perceived risk that the benefits of implementing best practices would accrue primarily to workers and the public good, rather than result in significant economic benefit to the industry itself. While the two are not incompatible, this lack of incentive can perpetuate release of toxic pollution despite knowledge of polluting activity, even when it is known to cause negative effects to human health, productivity, and planetary wellbeing.

Even in the absence of economic accounting for pollutants, given the associated mortality and productivity losses, investments in modern pollution reduction should be of global interest. In reality, funding towards reducing the impacts and exposures from modern pollution falls far below funding allocated to other major global health issues, even though the burden of disease may be significantly higher. While funding to other public health threats with high mortality rates are tracked annually by the Organization for Economic Cooperation and Development (OECD), funding towards modern pollution reduction is not. OECD tracks general spending on environmental protection, but this category is too broad to shed light on funding for modern pollution reduction. As no annual dataset tracks such investment, this research analyzes ODA funding allocated to reducing the human health impacts of modern pollution by reviewing data from bilateral and multilateral development agencies and international organizations.

This work can only be considered a preliminary attempt to quantify the ODA investments earmarked with the objective to reduce the negative human health impacts caused by modern pollution. There are substantive limitations to the analysis, partially detailed below, but results are sufficient to galvanize action to better monitor and track investments in modern pollution reduction. Beginning to quantify the funding disparity between well-funded public health problems compared to the lesser well-known but significantly underfunded problem of modern pollution will elevate the necessity of investing in programs to prevent and reduce modern pollution worldwide.

## II. Modern Pollution in a Modern World

The *Lancet* Commission defined pollution as “unwanted, often dangerous, material introduced into the Earth’s environment as the result of human activity that threatens human health harms ecosystems,” and is based on the European Union’s definition of pollution. Modern pollution, as defined by the *Lancet* report and the pollution.org platform is that “stemming from modern, industrial activities comprised of ambient air, chemical, and soil pollution” as opposed to traditional pollution defined as household air and water pollution [5]. This definition deemphasizes traditional household pollutants (indoor cook stoves, open defecation) as data from the *Lancet* showed that deaths from modern pollution are rising significantly, while deaths from traditional pollution are on the decline. Activities within the scope of modern pollution include those which release toxins into the environment (particulate matter, heavy metals, chemicals, and pesticides) and stem from agriculture (crop burning, heavy/overuse of inputs), extractive industries (artisanal mining), urbanization (vehicular exhaust) and industry (industrial waste, including artisanal activities). For the purpose of this research, funding was included if it targeted measures specifically focused on reducing the health impacts of pollution, and/or reducing pollutants known to negatively impact human health.

### Air pollution has some recent attention

Of the 9 million pollution-related deaths per year, ambient air pollution (not household air pollution) accounts for nearly 4.2 million deaths per year, with 91% of the world’s population living with air quality levels exceeding WHO limits [6]. Air pollution can be highly visible (smog, industrial emissions) and has been more well publicized in recent years. As a result, its profile has been significantly raised internationally, exemplified by the WHO adopting air pollution (but not other types of pollution) as a major risk factor for non-communicable diseases, and the increasing allocation of resources to combat air pollution, including the formation of the Clean Air Fund, and $413 million in new pledges in 2014 to the Clean Cooking Alliance [7,8].

Human activities that cause air pollution include fuel combustion, heat and power generation, industrial facilities, and municipal and agricultural waste. Pollutants generated by these activities include particulate matter (PM_1_ to PM_10_), black carbon, ground-level ozone (O_3_), nitrogen dioxide (NO_2_), sulphur dioxide (SO_2_), and nitrous oxide (NO_X_).

Health impacts of air pollution are well documented: heart disease, stroke, chronic obstructive pulmonary disease (COPD), lung cancer, and respiratory infections [6]. In 2013, the WHO’s International Agency for Research on Cancer classified PM_10_ and PM_2.5_ as causes of lung cancer due partially to the capability to penetrate the passageways of the lungs and enter into the bloodstream. Higher NO_2_ emissions are associated with increased medication expenditure and mortality rates [9]. Long-term exposure to particulate matter may impede cognitive performance, cause adolescent behavioral problems, lower birth weights for mothers living near high exposure zones, and lower child IQs as a result of impaired prenatal development [10,11,12,13]. Atmospheric pollution is associated with adverse effects on human health, even at levels below the permitted standards [14]. Emerging evidence on the impacts of air pollution on diabetes, dementia, and neurological development are growing, which, if substantiated, would greatly increase the burden of disease from ambient air pollution [1].

Measuring economic output from labor productivity, Zivin & Neidell (2012) [38] found that ozone levels (even below air quality standards) adversely impact agricultural productivity. T. Chang, Neidell, & Chang (2016) [33] found that PM_2.5_ negatively impacts agricultural productivity, and T. Chang, Zivin, Gross, & Neidell (2019) [34] suggest that high PM levels in China impact cognitive performance, where increases in the air pollution index lead to a decreased worker output. While labor productivity impacts may seem small ($0.41 per hour), T. Chang et al. (2016) [33] calculated an aggregate labor savings from PM_2.5_ reductions of nearly $19.5 billion. While the authors noted several limitations associated with this calculation, it serves as an early attempt to quantify the economic impacts of inaction. Similarly, a 2016 study funded by the World Bank estimated productivity losses of nearly 10% of GDP for China, 7.69% for India, and 8% in Sri Lanka and Cambodia [15].

### Governance

While non-state actors typically drive industrial emissions and absorb the bulk of the costs associated with adopting best practices to prevent pollution, the majority of the benefits of pollution prevention and control primarily accrue to the public sector (health improvements). While there is benefit to the private sector (reduced exposure risk, improved productivity and efficiency), this is not present in all industries, especially the informal sector. Thus, it is the State’s responsibility to deal with the social and political consequences and to bear the costs of enforcing pollution prevention and control. Likewise, where the polluter pays principle cannot be enforced, it falls to the State to implement mitigative and remediation measures.

In some countries, environmental policy is playing a more important role than 20 years ago, with politicians increasingly discussing environmental problems, such as climate change, as evidenced by the “Green New Deal” in the United States and increased attention to plastics pollution despite few quantifiable links to human health [16]. Pollution and health as a whole, especially that attributable to modern pollution, have not seen comparable political attention or public resource mobilization.

Transboundary pollution may have multinational consequences. Pollution does not stay in its country of origin, but moves through air, water, and product chains. Regulation is key, but weak enforcement in one area may have significant negative impacts in another [17]. For example, elevated levels of mercury are regularly found in tuna fish in European and US markets, yet the world’s largest source of anthropogenic mercury emissions emanates from artisanal gold mining in low- and middle-income countries – not coal fired powered plants. Mercury makes its way through the atmosphere and bioaccumulates in large pelagic fish, seals, and sharks. Transboundary pollution can also create strong potential for domestic and international conflict. Areas rendered inhabitable by pollution, which can exacerbate availability of clean water and soil already threatened by climate change-related drought or flooding, may cause population pressures and displacement.

### Modern pollution is more than air pollution

While ambient air and plastic pollution are more visible, pollution from heavy metals, chemicals, and wastes causes significant, invisible damage to human health. There are toxins in food, clothes, drinking water, and children’s toys [17]. From use of industrial wastewater contaminated with heavy metals to irrigate crops to use of lead and cadmium in toys and paint, the market system facilitates the global trade of contaminated goods. The *Lancet* Commission estimated deaths from water, soil, heavy metals, chemicals, and lead to be roughly a third of the 9 million deaths each year (2017). This is likely underestimated as the analysis does not include disability or death from environmental exposures from chemicals other than lead – which itself is also likely undercounted as it only considers ambient exposures to lead from leaded gasoline, phased out in the 1980–90’s. Thus, pesticides, chromium, mercury, endocrine disruptors, arsenic, asbestos and other known environmental toxicants, especially in soil, have not been sufficiently quantified to determine their global health impact.

However, data on the impacts of chemicals and heavy metals are growing. Research now shows public exposure to lead comes from unsafe processing and recycling of lead-acid batteries, pottery, paint, dyes, cookware, and contaminated food. In early 2019, the Institute of Health Metrics and Evaluation (IHME) increased the burden of disease globally from lead exposures to 1 million deaths in 2017 (also retroactively applied for previous years), up from the original 500,000 deaths cited in the *Lancet*, primarily due to better data in India alone [1,19]. In low-and middle-income countries, Attina and Trasande (2013) found the burden of childhood lead exposure amounts to 1.2% of global GDP for 2011, for a total of $977 billion international dollars.^1^ Ericson et al. (2018) [35] estimated the global burden of lead toxicity from lead-acid battery sites to be large (6 to 16.8 million exposed each year), with a range of 127,248 to 1.6 million DALYs.^2^ Research conducted for mercury exposure at artisanal small-scale gold mining sites estimated a global burden of disease ranging from 1.22 to 2.39 million DALYs [19].

Chronic pesticide exposures also pose significant concern. The failure to dispose of obsolete pesticides, and the availability and affordability of synthetic pesticides have led to increased exposure risks, especially in developing countries. Research on the health impacts of pesticides found in the food supply is inconclusive, but pesticide residues have been found on nearly 70% of the fresh produce tested by the United States Department of Agriculture [17]. Research on chronic exposure for agricultural workers provides evidence of negative health outcomes [20]. Cognitive impairment, fetal development, psychiatric disorders, and dementia have all been linked to pesticide exposures for agricultural workers and residents [21]. Chronic exposure has been linked to cancer, asthma, and diabetes [22]. Some pesticides are endocrine (hormone system) disrupters, which in addition to causing birth defects, cancer, and developmental disorders have long-term adverse effects on bee populations essential for pollination [23].

### Challenges of measuring pollution

Measuring pollution and its impacts is difficult as there is a non-linear relationship between levels of air pollution and health impacts [24]. This non-linear relationship also holds true for exposures to water and soil pollution, making isolation of different risk-factors challenging. It further provides no guarantee that any given reduction in pollution emissions will necessarily produce a reduction in illness, although there are well-documented examples where remediation of lead contaminated soil shows reductions in exposure risk and reductions in blood lead levels in affected children [25,26]. Other well-documented challenges include that atmospheric reactions occur when conditions change – temperature, humidity, wind, and existing pollution levels change readings from one area to another [24]. Placement of air quality monitors is not apolitical either. Grainger et al. (2017) [36] found evidence that environmental regulators in the US strategically place new ambient air pollution monitors, due in part to the high-costs borne by local government to reduce concentrations that violate US EPA standards. In developing countries, the lack of understanding of where and how pollution occurs is much more severe. Many poor countries have insufficient resources for data collection – limitations which likely reduce the accuracy of global estimates on the pollution related burden of disease [1].

### Tradeoffs

While productivity and health gains resulting from uses of chemicals cannot be dismissed, pollution can be controlled at the source, and is not a prerequisite for economic growth [1]. Although the proper use of pesticides does increase agricultural production and improve farmer incomes, the argument that the benefits of a polluting activity outweigh the costs is often erroneously applied to justify the unregulated, polluting economic activity. In reality, considerations for the lifelong negative impacts of exposure to pesticides or heavy metals (both to health and productivity) cannot be discounted. This health-wealth tradeoff is discussed by Benshaul-Tolonen (2018) [32] in an analysis of the impact of rapid industrial development in the mining sector in nine African countries. When a gold mine opens, the infant mortality rate decreases by over 50% within 10 kilometers and remains consistently lower throughout productive operations – but as a result of the increased economic activity, not because pollution controls were lacking. The long-term costs of unregulated polluting industry undermine the potency of any economic claims and further justify investments in pollution prevention and reduction.

## III. ODA Investments in Modern Pollution Reduction

### Methodology

Rigorous prioritization of different public health threats requires understanding how funds allocated to addressing each threat are able to reduce the threat’s impact (e.g., by reducing mortality rates). While calls for evidence-based policy evaluation have existed for decades, policy evaluation is hampered by lack of adequate record keeping for program expenditures and health outcomes [27,28]. In the absence of data on the changes in mortality associated with changes in funding provided to address a health threat, benefit-cost analysis cannot be conducted. Thus, decision-makers responsible for ODA allocations lack key information needed to not only evaluate effectiveness of interventions on public health (e.g. the “return on investment”), but also where significant investments are critical to reduce the burden of disease or what solutions are good investments. While there is evidence of the cost-effectiveness of some pollution interventions, for example Ericson, et al. (2018) [35] found that lead remediation in low- and middle-income countries is cost-effective according to WHO thresholds, overall comparisons of effectiveness of investments in modern pollution do not exist.

We introduce the metric of dollars spent per death caused by each health threat as a way of tracking the efficacy of expenditures. Under relatively restrictive assumptions (e.g., equal baseline mortality and expenditure figures across threats), a lower value of this metric for one health threat versus others is consistent with the finding that efforts to address this threat have a higher benefit-cost ratio than efforts to address other threats. Under these conditions, finding a lower value of dollars spent per death for a given health threat relative to other threats can be interpreted as evidence that more funding should be allocated to this threat.

The main objective of this research is to quantify the official development aid (ODA) allocated to combatting modern pollution from 2016 to 2018. Three main parameters defined the research: (1) determination of project inclusion, (2) the key search terms utilized, and (3) the countries or funding institutions included. Funding was included if it targeted measures that were specifically focused on reducing the health impacts of pollution, and/or reducing pollutants well-known to negatively impact human health. Relevant terms included the following: *pollution, industry, industrial, occupational, ambient, air, methane, chemical, soil, emission, ozone, carbon, lead, mercury, e-waste* (and variations thereof), *heavy metals, mining, pesticides, gold, crop burning*, and *agriculture*. The search was restricted to the 30 countries constituting the Development Assistance Committee (DAC) of the OECD and the European Union (Annex 1 lists those included in this research). This was primarily due to the fact that these represent the higher-income countries instrumental in driving the international development agenda and in their capacity to contribute large amounts of aid to globally pressing issues. We note that this approach focuses only on international aid flows and excludes domestic spending to combat pollution. This exclusion is most problematic in large transitioning economies like China and India that account for a large portion of the global deaths attributable to pollution and spend significant amounts – $2.6 billion in China in 2017 and $91 million for two years in India - to reduce air pollution [29,30]. The development aid contribution of China was not included.

To conduct the research, each country’s official international aid agency website and relevant publicly accessible online documents were searched for available project data, annual reports, and aid statistics using the search terms. Projects using the terms were analyzed to determine relevance to the scope of this research. Translation was conducted as needed using Google Translate. Investments in greenhouse gas emission reduction projects relating to clean energy, deforestation, and general emissions reductions were not included unless there was a clear aim to reduce particulate matter or a search term pollutant. The reason for this exclusion is that while there are overlaps between climate change and air pollution, carbon does not fall within the definition of pollution used by the *Lancet* Commission. Additionally, the scope of these projects was too large within the context of climate change to be specified as relating to the health impacts stemming from modern pollution. Funding to combat deforestation, for example, can be primarily to reduce carbon emissions, protect biodiversity, or fund enforcement mechanisms rather than seek to reduce or avoid open burning. While these investments may have positive health externalities, addressing health impacts were not the stated or primary goals. However, projects specifically mentioning reductions in agricultural emissions caused by crop burning were included because these have acute impact on surrounding community health. Projects related to indoor cooking, cleaner fuels, or water and sanitation were not included, as these are considered “traditional pollution”.

Contributions to international conventions and frameworks concerning pollutants and chemicals were considered, but these donations are reviewed separately because they do not track health performance, and it is impossible to capture to what extent these funds are used to reduce pollution. Included conventions/frameworks (herewith called “conventions”) include: The Montreal Protocol on Substances that Deplete the Ozone Layer, The Stockholm Convention on Persistent Organic Pollutants, The Chemicals and Waste and Pesticides special topic areas of the Global Environment Facility, the Basel Convention on the Control of Transboundary Movements of Hazardous Wastes and Their Disposal, the Rotterdam Convention, and the Minamata Convention. We provide a figure for convention contributions as a way to account for international funding allocated by governments. While the majority of data were available on the respective convention and program websites, agencies were contacted for more information as needed. The United Nations Development Program (UNDP), United Nations Environment Program (UNEP) Special Program on Chemicals and Waste Management Program, and the United Nations Industrial Development Organization (UNIDO) project data were also included.^3^

### Assumptions and Limitations

Several assumptions were made regarding the accuracy of reporting. Some countries provided full transparency via customized databases. Others provided general information on aid statistics and priority sectors via annual reports and summary documents, if at all. When project descriptions were not provided, it is probable relevant projects were unintentionally excluded. Limitations included the frequency, detail, and specificity with which countries update project data, or availability of data – Belgium and Republic of Korea had website problems and researcher inquiries resulted in no useful responses.

As the majority of reporting countries provided budgeted project data rather than funds allocated, budgeted data was used when both were provided. When appropriate, annual OECD exchange rates and price indices were used to convert values to constant 2017 dollars.

Last but not least, using deaths as a measure for human impact itself is problematic. While easily quantified, deaths represent the lower bound of welfare impacts from pollution. This stems from many factors, including that health impacts of pollution itself are difficult to isolate and that exposure to pollution causes diseases which individuals can live with (albeit with compromised quality of life, increased health care costs, or lost/reduced productivity). Further, pollution is typically a risk factor for death, and rarely attributed as a cause of death. To the contrary, contagious diseases or those transmitted through vectors (malaria) are verifiable causes of death, and investments in prevention promote herd immunity, whereby vaccination or protection for one individual or group reduces likelihood of transmission and death for the entire population. For pollution, reduction of exposure risk and levels of contamination are the metric.

## IV. Results

For all DAC countries, we find the average DAC contribution from 2016 to 2018 to be $57,396,492 for modern pollution projects, and $74,997,311 for total project spending (Table 1).

**Table 1:** Contributions to modern pollution reduction, 2016–2018.

	2016	2017	2018	Average

**DAC Countries**	$64,460,824	$49,953,444	$57,775,207	$57,396,492
**UNDP, UNEP, UNIDO**	$14,503,011	$13,221,824	$25,077,623	$17,600,819
**TOTALS**	**$78,963,836**	**$63,175,268**	**$82,852,830**	**$74,997,311**
**Conventions**	$279,840,345	$279,420,131	$301,231,997	$286,830,824

All values are constant 2017 USD.

Project data from UNDP, UNEP, and UNIDO are listed separately. Their contributions are non-trivial but are covered neither in other contributions nor country level data. For UNEP, care was taken to ensure contributions to conventions (from the UNEP Special Program on Chemicals and Waste Management Program) did not result in double counting. Convention contributions are shown separately, as it is difficult to determine what amount of spending could be considered to impact health. As a result, these numbers overestimate funding allocated to projects alone. Annex 2 (Excel) includes all projects, data, and calculations associated with the tables in Section IV. Data for project and convention contributions by country is available in Table 2: Funding breakdowns by country.

**Table 2:** Funding breakdowns by country.

	Projects Funding Modern Pollution Reduction						Convention Contributions					

		2016		2017		2018		2016		2017		2018

Australia	$	130,738	$	90,000	$	102,015	$	8,069,491	$	7,847,443	$	9,542,880
Austria	$	27,930	$	7,043	$	–	$	4,316,044	$	4,269,765	$	4,535,746
Belgium	$	–	$	–	$	–	$	6,127,054	$	6,069,176	$	5,964,270
Canada	$	11,844,767	$	10,244,563	$	13,308,009	$	15,480,487	$	15,129,393	$	16,546,001
Czech Republic	$	–	$	–	$	–	$	1,098,076	$	1,056,066	$	1,132,329
Denmark	$	7,181,301	$	8,200,000	$	245,935	$	4,457,921	$	4,457,398	$	3,180,054
European Union	$	3,667,540	$	3,682,669	$	3,702,346	$	302,681	$	235,251	$	701,752
Finland	$	–	$	–	$	–	$	4,537,948	$	4,519,158	$	3,770,820
France	$	586,614	$	993,858	$	824,872	$	23,363,526	$	23,161,033	$	25,143,814
Germany	$	7,874,985	$	8,879,731	$	9,335,435	$	33,056,975	$	32,682,605	$	36,805,415
Greece	$	–	$	–	$	–	$	1,390,137	$	1,367,039	$	1,213,907
Hungary	$	–	$	–	$	–	$	600,663	$	569,957	$	417,371
Iceland	$	–	$	–	$	–	$	64,010	$	57,853	$	62,133
Ireland	$	27,634	$	–	$	–	$	1,227,395	$	1,203,333	$	1,181,935
Italy	$	66,630	$	117,564	$	523,326	$	13,914,318	$	13,760,323	$	14,080,647
Japan	$	130,472	$	125,706	$	153,250	$	44,950,597	$	45,214,059	$	51,462,813
Korea	$	–	$	–	$	–	$	574,819	$	558,180	$	598,545
Luxembourg	$	450,505	$	450,757	$	–	$	405,329	$	399,187	$	409,266
The Netherlands	$	2,746,575	$	111,424	$	767,523	$	7,661,947	$	7,643,181	$	8,068,548
New Zealand	$	237,737	$	–	$	–	$	792,678	$	769,383	$	1,052,946
Norway	$	3,236,157	$	898,851	$	758,750	$	4,615,661	$	4,571,511	$	5,034,597
Poland	$	57,986	$	–	$	–	$	2,085,462	$	1,973,421	$	2,183,388
Portugal	$	–	$	–	$	–	$	1,061,093	$	1,015,636	$	1,009,884
Slovak Republic	$	–	$	–	$	–	$	374,388	$	366,401	$	412,010
Slovenia	$	–	$	–	$	–	$	445,428	$	438,863	$	462,085
Spain	$	–	$	–	$	–	$	7,987,513	$	7,879,877	$	7,064,865
Sweden	$	6,837,232	$	5,077,562	$	7,065,239	$	9,600,862	$	9,583,537	$	12,012,278
Switzerland	$	665,566	$	680,416	$	695,156	$	7,168,001	$	7,496,425	$	8,625,768
United Kingdom	$	13,848,348	$	4,817,539	$	9,836,400	$	22,063,838	$	22,503,252	$	24,388,878
United States	$	4,842,105	$	5,575,761	$	10,456,951	$	52,046,001	$	52,621,425	$	54,167,052
**Organization**												
UNDP, UNEP, UNIDO	$	14,503,011	$	13,221,824	$	25,077,623	$	–	$	–	$	–
**Totals**	$	78,963,836	$	63,175,268	$	82,852,830	$	279,840,345	$	279,420,131	$	301,231,997

Deaths from modern pollution are taken from The *Lancet* Commission report GBD study best estimates (2015), excluding values for water and household air pollution, as projects addressing these issues were not included in the scope of this study (Table 3). These 2015 numbers were used for all future years.

**Table 3:** Burden of disease (deaths) from Modern Pollution.

	GBD Study (2015)	Modern Deaths^2^	Pollution

**Air (Total)**	**6.5 (5.7–7.3)**	**3.8**	
Household Air	2.9 (2.2–3.6)	*–*	
Ambient	4.2 (3.7–4.8)	*4.2* (*3.7 – 4.8*)	
Particulate	0.3 (0.1–0.4)	*0.3* (*0.1 – 0.4*)	
Ambient Ozone			
**Water (Total)**	**1.8 (1.4–2.2)**	*–*	
Unsafe	0.8 (0.7–0.9)		
Sanitation	1.3 (1.0–1.4)		
Unsafe Source			
**Occupational**	**0.8 (0.8–0.9)**	**0.8 (0.8–0.9)**	
Carcinogens	0.5 (0.5–0.5)	0.5 (0.5–0.5)	
Particulates	0.4 (0.3–0.4)	0.4 (0.3–0.4)	
**Soil, Heavy Metals, and Chemicals**	**1.0 (0.2–0.8)**	**0.5 (0.2–0.8)**	
**Lead**	**0.5 (0.2–0.8)**	**1.0**^3^	
**Total Estimated Deaths (Millions)**	**9.0**	**5.8**	

^1^ Note that the totals for air pollution, water pollution, and all pollution are less than the arithmetic sum of the individual risk factors within each of those categories because these have overlapping contributions – eg, household air pollution also contributes to ambient air pollution and vice versa. ^2^ To be conservative, the lower end of the 95% confidence interval was used. ^3^ IHME retroactively updated lead deaths in 2017 to 1 million. This change has been reflected here. (IHME, 2019) [18].

A comparison with global investment in other public health areas illustrates the insignificance of $72 million annually for modern pollution reduction, considering its burden of disease. Table 4 compares spending in three top public health areas of global concern (malaria, HIV/AIDS, and tuberculosis) and general environmental protection.^4^ Funding data for these was obtained from OECD’s Query Wizard for International Development Database. Deaths for malaria, HIV/AIDS, and tuberculosis are taken from IHME’s Database [18,31].

**Table 4:** Development funding allocated to major public health risks.

	Deaths/Year			Funding/Year^1^ (Constant 2017 USD)			Funding Allocated/Death (Constant 2017 USD)		

	2016	2017	2018^2^	2016	2017	2018^2^	2016	2017	2018

HIV/AIDS	625,834	619,827	619,827	$782,248,303	$630,353,038	$630,353,038	$1,250	$1,017	$1,017
Malaria	1,041,798	954,492	954,492	$171,110,670	$114,290,584	$114,290,584	$164	$120	$120
Tuberculosis	1,200,508	1,183,672	1,183,672	$228,646,145	$290,761,100	$290,761,100	$190	$246	$246
Modern Pollution	5,800,000	5,800,000	5,800,000	$78,963,836	$63,175,268	$82,852,830	**$14**	**$11**	**$14**
Convention Contributions				$279,840,345	$279,420,131	$301,231,997			
Modern pollution + conventions				$358,804,180	$342,595,400	$384,084,827	**$62**	**$59**	**$66**
Gen. Environmental Protection				$3,692,276,341	$3,637,860,474	$3,637,860,474			

^1^ Constant 2017 USD. ^2^ Deaths are not available yet for 2018, 2017 is used as a conservative estimation for 2018.

In 2016, we calculate an average investment of $14/death for modern pollution, compared with $1,250/death for malaria, $190/death for tuberculosis, and $165/death for HIV/AIDS using only DAC spending reported by the OECD (Table 4). While deaths from modern pollution are higher than malaria by 900%, OECD spending for modern pollution amounts to 10% of that allocated to malaria. Total ODA spending on the environment in general, however, *is* considerably larger than spending in three listed public health areas. This suggests that there may not be a disconnect in sectors where funding is allocated, but rather a mis-prioritization of modern pollution and the need to highlight it as a cross-sectoral problem. This underlines the need for funding to be allocated *specifically* to modern pollution reduction, as it is not sufficiently addressed within either the health or environment sectors in a manner that reflects the severity of the public health burden caused by toxic pollution exposure.

## V. Conclusions

While the limitations, difficulties, and assumptions made for this analysis are myriad, the implications are clear. ODA investment to reduce negative human health impacts from exposure to modern pollutants and toxicity is lower than funding for other health concerns that have smaller impacts on mortality. Deaths from malaria, HIV/AIDS, and tuberculosis are now far lower than those estimated from modern pollution – in large part due to the level of aid that has been invested to solve these critical public health problems over recent decades. While the media and popular opinion is starting to emphasize the ominous impacts of modern pollution on human health, the funding sphere has been slow to respond.

This research seeks to highlight the funding disparity between select key threats to global health. While there are limitations to this research, the hope is that it contributes to two things: (1) a call for more transparency and tracking of funding allocated to modern pollution and (2) that beginning to quantify the funding disparity will lead to serious calls to international donors to narrow this gap. The difficulties of measuring and documenting pollution are known, and political and economic realities often outweigh good environmental policy in seeking to monitor and reduce pollution. However, effective solutions exist for reducing the impacts of modern pollution on human health. This research underlines the severe funding gap and the merits of investing, prioritizing, and tracking ODA spending on pollution, and raises an urgent alarm about the failure of DAC countries to adequately respond to this urgent public health crisis.

## Additional Files

The additional files for this article can be found as follows:

10.5334/aogh.2633.s1 Annex 1. Annex 1 provides a list of all the countries (plus the European Union) provided in this research. All countries are OECD Development Assistance Committee (DAC) Member States.

10.5334/aogh.2633.s2 Annex 2. Annex 2 is a Microsoft Excel document containing all projects, data, and calculations associated with the tables and results in Section IV.

## Footnotes

An international dollar would buy in the cited country a comparable amount of goods and services a U.S. dollar would buy in the United States. This term is often used in conjunction with Purchasing Power Parity (PPP) data.

One Disability-Adjusted Life Year (DALY) can be thought of as one lost year of “healthy” life. The sum of these DALYs across the population, or the burden of disease, can be thought of as a measurement of the gap between current health status and an ideal health situation where the entire population lives to an advanced age, free of disease and disability (World Health Organization, 2014). [37].

Data from the Strategic Approach to International Chemicals Management (SAICM) was not included as the last funding round of SAICM was 2015.

General environmental protection includes funding towards environment protection, environmental policy and administrative management, biosphere protection, bio-diversity, site preservation, environmental education/training, environmental research (OECD, 2019) [31].
