# Supplementary material for: Rethinking Aid Allocation: Analysis of Official Development Spending on Modern Pollution Reduction
Source: Ann Glob Health. 2019 Nov 7;85(1):132. doi: 10.5334/aogh.2633 (PMC6838771; doi:10.5334/aogh.2633)
Supplement: Annex 1. — Annex 1 provides a list of all the countries (plus the European Union) provided in this research. All countries are OECD Development Assistance Committee (DAC) Member States. [file agh-85-1-2633-s1.pdf]

Annex I

**Country/Organization**	**Aid Agency(s) Included**
Australia	Department of Foreign Affairs and Trade (DFAT)
Austria	Austria Development Agency (ADA)
Belgium	Belgian Development Agency (Enabel)
Canada	Global Affairs Canada
Czech Republic	Ministry of Foreign Affairs of the Czech Republic
Denmark	Danish Development Corporation
European Union	EU International Cooperation and Development
Finland	Ministry of Foreign Affairs of Finland
France	Agency Française de Development (AFD); Fonds Français Pour l'Environnment Mondial (FFEM)
Germany	Deutsche Gesellschaft für Internationale Zusammenarbeit (GIZ)
Greece	Hellenic Aid, Ministry of Foreign Affairs of Greece
Hungary	Ministry of Foreign Affairs and Trade
Iceland	International Development Cooperation (Iceida)
Ireland	Irish Aid
Italy	Italian Agency for Development Cooperation (AICS)
Japan	Japan International Cooperation Agency (JICA), Environmental Restoration and Conservation Agency (ERCA)
Korea	Korean International Cooperation Agency (KOICA)
Luxembourg	LuxDev
The Netherlands	Government of Netherlands Development Cooperation

New Zealand	New Zealand Foreign Affairs and Trade
Norway	The Norwegian Agency for Development Cooperation (NORAD)
Poland	Polish Aid
Portugal	Ministry of Foreign Affairs
Slovak Republic	Slovak Aid
Slovenia	International Development Cooperation of Slovenia
Spain	Spanish Agency for International Development Cooperation (Aecid)
Sweden	Swedish Aid
Switzerland	Swiss Development Cooperation
United Kingdom	Department for International Development (DFID)
United States	United States Agency for International Development (USAID)
